# Measurement of hepatic insulin sensitivity early after the bypass of the proximal small bowel in humans

**DOI:** 10.1002/osp4.76

**Published:** 2016-12-29

**Authors:** A. D. Miras, R. Herring, A. Vusirikala, F. Shojaee‐Moradi, N. C. Jackson, S. Chandaria, S. N. Jackson, A. P. Goldstone, N. Hakim, A. G. Patel, A. M. Umpleby, C. W. Le Roux

**Affiliations:** ^1^ Section of Investigative Medicine, Division of Diabetes, Endocrinology and Metabolism Imperial College London London UK; ^2^ CEDAR Centre Royal Surrey County Hospital Guildford Surrey UK; ^3^ University of Glasgow Glasgow UK; ^4^ Diabetes and Metabolic Medicine, Faculty of Health and Medical Sciences University of Surrey Guildford UK; ^5^ National Obesity Forum UK; ^6^ Centre for Neuropsychopharmacology, Computational, Cognitive and Clinical Neuroimaging Laboratory, Division of Brain Sciences Imperial College London London UK; ^7^ Faculty of Medicine, Department of Surgery and Cancer Imperial College London London UK; ^8^ Hepatobiliary and minimal access surgery King's College Hospital NHS Foundation Trust London UK; ^9^ Diabetes Complications Research Centre, UCD Conway Institute University College Dublin Dublin Ireland

**Keywords:** Caloric restriction, duodeno‐jejunal bypass liner, endobarrier, gastric bypass

## Abstract

**Objective:**

Unlike gastric banding or sleeve gastrectomy procedures, intestinal bypass procedures, Roux‐en‐Y gastric bypass in particular, lead to rapid improvements in glycaemia early after surgery. The bypass of the proximal small bowel may have weight loss and even caloric restriction‐independent glucose‐lowering properties on hepatic insulin sensitivity. In this first human mechanistic study, we examined this hypothesis by investigating the early effects of the duodeno‐jejunal bypass liner (DJBL; GI Dynamics, USA) on the hepatic insulin sensitivity by using the gold standard euglycaemic hyperinsulinaemic clamp methodology.

**Method:**

Seven patients with obesity underwent measurement of hepatic insulin sensitivity at baseline, 1 week after a low‐calorie liquid diet and after a further 1 week following insertion of the DJBL whilst on the same diet.

**Results:**

Duodeno‐jejunal bypass liner did not improve the insulin sensitivity of hepatic glucose production beyond the improvements achieved with caloric restriction.

**Conclusions:**

Caloric restriction may be the predominant driver of early increases in hepatic insulin sensitivity after the endoscopic bypass of the proximal small bowel. The same mechanism may be at play after Roux‐en‐Y gastric bypass and explain, at least in part, the rapid improvements in glycaemia.

## Introduction

Bariatric surgery is the most effective treatment for glucose control in patients with type 2 diabetes mellitus with obesity (e.g. 1). Unlike gastric banding procedures, intestinal bypass procedures, Roux‐en‐Y gastric bypass in particular, lead to rapid improvements in glycaemia early after surgery 2. Duodeno‐jejunal bypass surgery in non‐obese streptozotocin‐induced diabetic rats reduced plasma glucose and hepatic insulin sensitivity within 2 d of surgery, independent of changes in caloric intake 3. In addition, we and others have previously shown that 1 week after the endoscopic implantation of the duodeno‐jejunal bypass liner (DJBL) in humans, fasting and post‐prandial glucose – but not insulin – concentrations are significantly reduced 4, 5. In these studies, markers of hepatic insulin sensitivity – but not insulin secretion – improved early after the procedure and before substantial weight loss has taken place. The results of these animal and human studies have therefore given rise to the hypothesis that the bypass of the proximal small bowel may have weight loss and even caloric restriction‐independent effects on hepatic insulin sensitivity.

However, even in the absence of any intestinal interventions, caloric restriction *per se* also improves glucose homeostasis and hepatic insulin sensitivity 6, and the human DJBL studies did not control for this important confounder. In this first human mechanistic study, we controlled for the effects of caloric restriction and examined the early effects of the DJBL on the insulin sensitivity of hepatic glucose production (HGP) by using the gold standard euglycaemic hyperinsulinaemic clamp methodology.

## Methods

The trial protocols were approved by the West London 2 Research Ethics Committee (reference 11/LO/0322). All participants gave written informed consent, and the trials were performed according to the principles of the Declaration of Helsinki.

Seven patients with obesity who were due to undergo treatment with the DJBL were recruited. This medical device is made from a non‐permeable fluoropolymer and is inserted endoscopically to line the first 60 cm of the small bowel. Functionally, this prevents food from having contact with the mucosa of the proximal small bowel, whilst simultaneously, bile salts flow undiluted on this mucosa before mixing with food in the jejunum. The insulin sensitivity of HGP was measured by using low‐dose euglycaemic hyperinsulinaemic clamps that were performed on three occasions: (i) at baseline – visit 1; (ii) 1 week after a low‐calorie liquid diet (Fortisip^®^ compact, 1,500 kCal d^−1^) – visit 2; and (iii) 1 week after insertion of the DJBL whilst on the same low‐calorie diet (LCD) – visit 3. Dietary adherence was assessed with food diaries.

Patients attended in the fasted state. After cannulation, a primed continuous infusion of [6, 6‐^2^H_2_] glucose (170 mg; 1.7 mg/min) was commenced. Once a steady state of enrichment with the stable isotopes was achieved, five baseline samples were taken between 100 and 120 min for measurement of the glucose enrichment. At 120 min, a one‐step low‐dose euglycaemic hyperinsulinaemic clamp was initiated. Insulin was infused at 0.3 mU/kg^−1^ · min^−1^ (Actrapid, Novo Nordisk, Copenhagen, Denmark) for 120 min to measure the insulin sensitivity of endogenous glucose production, which is predominantly a measure of HGP. Plasma glucose concentration was maintained at fasting levels by using a variable infusion of 20% dextrose spiked with [6, 6‐^2^H_2_] glucose (8 mg/g). Blood samples were taken every 10 min, with blood glucose measured immediately by the glucose oxidase method by using a YSI biochemistry analyzer (YSI Life Sciences, Yellow Springs, Ohio). The isotopic enrichment of plasma glucose was measured by gas chromatography‐mass spectrometry on an Agilent Technologies 5975C inert Xl EI/CI MSD system. Glucose concentrations were measured on the Mira autoanalyser by using the glucose PAP assay (Horiba). HGP and glucose disposal rate (*R*_d_) were calculated, using the model proposed by Steele 7 modified for the inclusion of stable isotopes 8.

Glucose‐lowering therapy did not change after the interventions in order to minimize the effects of this important confounder.

Data are described as mean ± standard deviation. Statistical comparisons were made by using a repeated measures one‐way anova with a *post hoc* Newman–Keuls multiple comparison test on PRISM^®^ version 5.

## Results

The seven patients were all European Caucasians (Table 1). Six of the patients had type 2 diabetes treated with metformin (*n* = 5), liraglutide (*n* = 2), pioglitazone (*n* = 1) and insulin (*n* = 3). The patients reported good overall compliance with the 1,500 kCal liquid LCD in their food diaries, apart from 1 to 3 d after the insertion of the DJBL when they reported consuming 1,200–1,500 kCal of the liquid diet. Hypoglycaemia was not reported by any of our participants.

**Table 1:** Participant characteristics/data at baseline and during the course of the study

	Visit 1 baseline	Visit 2 post LCD	Visit 3 post DJBL	anova *p*‐value	Pairwise comparisons
Age (years)	52 ± 12	—	—	—	—
Gender (M/F)	5/2	—	—	—	—
HbA1c (mmol/mol) (%)	53 ± 17 7.2 ± 1.6	—	—	—	—
BMI (Kg m^−2^)	48.5 ± 7.9	47.1 ± 7.6	45.7 ± 7.8	<0.0001	Visit 1 vs. 2 *p* < 0.01
					Visit 2 vs. 3 *p* < 0.01
					Visit 1 vs. 3 *p* < 0.001
Weight (Kg)	141.9 ± 26.5	138.3 ± 26.1	134.0 ± 26.3	<0.0001	Visit 1 vs. 2 *p* < 0.01
					Visit 2 vs. 3 *p* < 0.01
					Visit 1 vs. 3 *p* < 0.001
Absolute body weight loss vs. baseline (Kg)	‐	3.6 ± 1.7	7.9 ± 3.3	n/a	—
% body weight loss vs. baseline	‐	2.5 ± 1.1	5.7 ± 2.5	n/a	—
Fasting glucose (mmol L^−1^)	8.8 ± 3.1	7.0 ± 2.4	7.4 ± 1.7	0.04	Visit 1 vs. 2 *p* < 0.05
HGP – before clamp (µmol · kg^−1^ · min^−1^)	9.3 ± 0.7	8.8 ± 1.5	8.9 ± 1.7	0.52	—
HGP – during clamp (µmol · kg^−1^ · min^−1^) % *r* reduction from basal	4.3 ± 1.3	3.4 ± 1.4	3.8 ± 1.1	0.047	Visit 1 vs. 2 *p* < 0.05
	54.5 ± 12.4	61.9 ± 12.3	56.8 ± 9.4	0.13	—
Glucose Rd – during clamp(µmol · kg^−1^ · min^−1^)	8.9 ± 1.5	8.7 ± 1.3	8.5 ± 1.0	0.74	—
Insulin clearance (L • kg^−1^ • min^−1^)	69.3 ± 17.9	68.2 ± 20.9	71.6 ± 24.0	0.72	—
Insulin – before clamp (pmol L^−1^)	99.8 ± 42.7	91.5 ± 36.2	78.4 ± 30.7	0.09	—
Insulin – during clamp (pmol L^−1^)	233.1 ± 62.9	234.8 ± 68.0	220.3 ± 74.9	0.55	—
Glucose – during clamp (mmol L^−1^)	5.15 ± 0.46	5.21 ± 0.57	5.20 ± 0.42	0.85	‐
CRP (mg L^−1^)	6.4 ± 2.4	8.8 ± 4.0	16.9 ± 7.5	0.005	Visit 2 vs. 3 *p* < 0.05
					Visit 1 vs. 3 *p* < 0.01

Data are described as mean ± standard deviation. Statistical comparisons were made by using repeated measures one‐way anova with *post hoc* Newman–Keuls corrected pairwise comparisons which are shown when statistically significant. ‘Before’ and ‘during’ refer to the 20 min before and the last 30 min of the euglycaemic hyperinsulinaemic clamp respectively. Abbreviations: BMI, body mass index; CRP, c‐reactive protein; DJBL, duodenal‐jejunal bypass liner; HbA1c, glycated haemoglobin; HGP, hepatic glucose production; LCD, low‐calorie diet; n/a, not applicable; Rd, rate of disposal.

**Figure 1:**
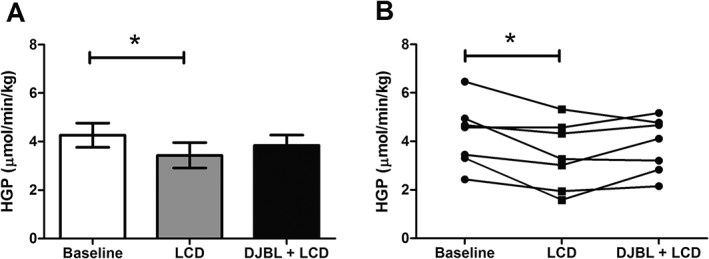
Group and individual responses in hepatic glucose production to insulin before and after a liquid low‐calorie diet and implantation of the duodeno‐jejunal bypass liner. Bars represent group mean ± standard deviation (A) and plots of individual responses (B) of hepatic glucose production at baseline, after 1 week on a liquid low‐calorie diet and 1 week after the implantation of the duodeno‐jejunal bypass liner. Comparisons were performed by using a one‐way repeated measures anova.

Hepatic glucose production before the clamp and glucose and insulin concentrations during the clamp were similar between the three visits (Table 1). HGP during the clamp and fasting plasma glucose were significantly reduced on visit 2 but not significantly reduced any further on visit 3 (Figure 1, Table 1). C‐reactive protein, a marker of inflammation, increased significantly on visit 3 compared with baseline.

## Discussion

In this mechanistic study, the endoscopic bypass of the proximal small bowel with a DJBL device did not reduce hepatic insulin sensitivity beyond the improvements achieved with caloric restriction.

Previous human DJBL studies controlled for the effects of weight loss but not for the reduction in caloric intake that takes place after the procedure 4, 5. The effects of caloric restriction were controlled for in an animal experiment where metabolic markers were compared between a group of Zucker diabetic fatty rats implanted with the DJBL and a group that was paired‐fed to the DJBL rats 9. Indeed, there were no significant differences in markers of hepatic insulin sensitivity (assessed indirectly by fasting glucose and insulin concentrations) between the groups, a finding which is consistent with the results of this current study in humans.

The novelty of our study lies in that we largely controlled for the effects of caloric restriction by asking our patients to consume the same low‐calorie liquid diet both before *and* after the bypass of their proximal small bowel. Between visits 1 and 2, and whilst on the low‐calorie liquid diet, our patients exhibited the expected improvement in hepatic insulin sensitivity as reflected by the significantly lower HGP. The small amount of weight loss after the DJBL implantation suggests that the patients were still in a negative energy balance secondary to reduced calorie intake. Even so, this did not translate to an additional reduction in HGP. We cannot exclude that the rise in inflammatory markers after the procedure may have masked and counteracted any effects that the DJBL may have on insulin sensitivity; nevertheless, these effects, if any, are likely to be of small physiological and clinical significance.

Our study is limited by the small number of participants and the lack of a control group, and therefore our findings will need to be confirmed by larger randomized controlled trials (e.g. ISRCTN30845205). As already mentioned, whilst our participants were instructed to consume the same amount of the LCD both before and after the DJBL implantation, this may not have been fully possible post‐procedure due to the expected side effects of abdominal pain and nausea. Nevertheless, the reduction in caloric intake was reported to be small and so was the weight loss achieved.

## Conclusion

Our findings suggest that caloric restriction may be the predominant driver of early increases in hepatic insulin sensitivity after the endoscopic bypass of the proximal small bowel. The same mechanism may be at play after Roux‐en‐Y gastric bypass and explain, at least in part, the rapid improvements in glycaemia observed early after surgery.

## Conflict of Interest Statement

None relevant to this manuscript for any of the authors.

## Funding

The Section of Endocrinology and Investigative Medicine is funded by grants from the MRC, BBSRC and NIHR, an Integrative Mammalian Biology (IMB) Capacity Building Award, a EuroCHIP grant (FP7‐HEALTH‐2009‐241592) and is supported by the NIHR Biomedical Research Centre Funding Scheme. The views expressed are those of the author(s) and not necessarily those of the NHS, the NIHR or the Department of Health. Alexander D. Miras has received funding from an MRC Clinical Training Fellowship, an MRC Centenary Award and an NIHR Clinical Lectureship. Carel W. le Roux has received funding from the Science Foundation Ireland (12/YI/B2480) and the Moulton Foundation UK.
